# Development and internal validation of a risk prediction model for gastric precancerous lesions integrating *Helicobacter pylori* serologic typing and host susceptibility polymorphisms

**DOI:** 10.1097/MD.0000000000049606

**Published:** 2026-07-03

**Authors:** Huiling Yu, Ping Li, Shujun Gong, Jingwen Zhao, Kui Jiang

**Affiliations:** a Department of Gastroenterology and Hepatology, Tianjin Medical University General Hospital, Tianjin Institute of Digestive Diseases, Tianjin Key Laboratory of Digestive Diseases, Tianjin, China; b Department of Gastroenterology, Affiliated Hospital of Hebei University, Baoding, Hebei, China.

**Keywords:** gastric precancerous lesions, *Helicobacter pylori* typing, risk prediction model, risk factors, susceptibility gene

## Abstract

This study aimed to develop and internally validate a clinical risk prediction model for precancerous lesions of gastric cancer (PLGC) in *Helicobacter pylori (H. pylori*)–positive individuals by integrating *H. pylori* strain typing, serological gastric function markers, lifestyle factors, and host susceptibility polymorphisms. A total of 82 *H. pylori*–positive outpatients who underwent gastroscopy with histopathology between July 2020 and December 2021 were enrolled and categorized into a PLGC group (n = 38; chronic atrophic gastritis, intestinal metaplasia, and/or dysplasia) and a non-PLGC control group (n = 44). Demographic, clinical, dietary, and lifestyle information was collected using standardized questionnaires. Fasting blood samples were obtained for pepsinogen I (PG I), pepsinogen II (PG II), pepsinogen I/II ratio (PGR), gastrin-17 (G-17), and serologic *H. pylori* typing (Type I vs Type II), and 4 gastric cancer susceptibility loci (PSCA rs2976392, PLCE1 rs2274223, PRKAA1 rs59133000, and MUC1 rs4072037) were genotyped using TaqMan assays. Multivariable logistic regression identified older age and Type I *H. pylori* infection as independent risk factors, whereas higher PG I, higher PGR, and frequent whole-grain intake were protective. Susceptibility loci were not retained in the final model. The derived model showed adequate calibration (Hosmer–Lemeshow *P* = .426) and strong discrimination (AUC = 0.969), with an optimal cutoff of 0.403 yielding 94.7% sensitivity and 93.2% specificity; bootstrap validation indicated stable performance. This model may support risk stratification and targeted surveillance among *H. pylori*–positive patients.

## 1. Introduction

Gastric cancer is the fifth leading cause of cancer and the fourth leading cause of cancer death worldwide.^[1]^ Gastric cancer progresses through a well-recognized precancerous cascade, beginning with normal gastric mucosa and advancing through chronic superficial gastritis, chronic atrophic gastritis, intestinal metaplasia, and dysplasia before culminating in gastric cancer.^[2]^ Precancerous lesions of gastric cancer (PLGC) refers to a kind of pathological and histological change of gastric mucosa that is prone to cancer. The PLGC we studied included chronic atrophic gastritis, intestinal metaplasia, and dysplasia. Early intervention during these precancerous stages can substantially reduce the incidence of gastric cancer. Thus, early screening of patients with PLGC, regular follow-ups, and timely intervention can enhance early diagnosis and prevent the occurrence of gastric cancer. However, widespread gastroscopy screening has not been implemented due to low acceptance rates stemming from patient fear and discomfort associated with the procedure. To address this challenge, establishing a risk model for identifying high-risk groups could facilitate early diagnosis and treatment of gastric cancer without relying solely on gastroscopy. In light of this situation, we analyzed risk factors for PLGC and developed a screening model for high-risk PLGC populations.

*Helicobacter pylori (H. pylori*) is a well-recognized carcinogenic bacterium that plays a crucial role in the development of PLGC and gastric cancer itself. Based on their virulence factors, *H. pylori* strains are categorized into Type I and Type II. Type I strains produce significant virulence proteins, notably cytotoxin-associated gene A (CagA) and vacuolating cytotoxin A (VacA), whose genetic diversity is closely linked to an increased risk of gastric cancer and associated precancerous conditions.^[3]^ In addition, host genetic factors considerably affect gastric carcinogenesis. single-nucleotide polymorphisms, the most common type of genetic variation, can directly or indirectly affect gene expression, providing crucial information for identifying high-risk groups for gastric cancer. Prostate stem cell antigen (PSCA) gene polymorphisms and increased risk of gastric cancer were reported in a two-stage genome-wide association study (GWAS) in Korean and Japanese populations.^[4]^ Especially for PSCA, the gene locus rs2294008 C>T and rs2976392 G>A polymorphisms were significantly associated with an increased risk of gastric cancer in the Chinese population.^[5]^ Although numerous gastric cancer susceptibility loci have been widely reported, few studies have incorporated both *H. pylori* strain typing and genetic susceptibility genes into the predictive models for PLGC occurrence.

In the present study, we analyzed the general clinical characteristics, laboratory findings, *H. pylori* strain types, and gene polymorphisms of *H. pylori*-positive patients. Using multivariate regression analysis, we constructed a clinical prediction model for PLGC based on *H. pylori* typing. Our aim was to assess the risk of PLGC in *H. pylori*-positive individuals and provide a scientific basis for the screening of patients with PLGC and the early diagnosis of gastric cancer.

## 2. Information and methods

### 2.1. General information

This study was approved by the Ethics Committee of Tianjin Medical University General Hospital. This study included 82 *H. pylori*-positive patients who attended the outpatient clinic at Tianjin Medical University General Hospital between July 2020 and December 2021. All patients had confirmed gastroscopic pathology and met the following inclusion criteria: aged between 18 and 80 years, regardless of sex; positive *H. pylori* status, confirmed using the ^13^C urea breath test; underwent gastroscopy and pathological examination within the preceding 3 months; did not use antibiotics in the past 4 weeks or proton pump inhibitors in the past 2 weeks; and agreed to participate in the study, providing consent for their blood to be sampled as well as signed informed consent. Exclusion criteria were as follows: presence of malignant tumors in other organ systems, known cardiovascular or cerebrovascular diseases such as coronary artery disease and cerebral infarction, hepatic or renal insufficiency, severe mental illness, pregnancy or lactation, and other conditions that could significantly affect the study results. Based on these criteria, the patients were divided into 2 groups: a case group, comprising 38 patients (with gastric precancerous lesions), and a control group, consisting of 44 patients (without gastric precancerous lesions).

The comprehensive clinical data were collected for each patient. Demographic information included age, sex, and education level. We recorded family history of tumors and assessed dietary habits, including consumption frequency of hot, fried, spicy, or high-salt foods; irregular eating patterns; and intake frequency of coarse cereals, vegetables, fruits, onions, ginger, garlic, pickles, and dairy products. Frequent whole-grain intake was defined as consumption more than twice per week, according to previous dietary epidemiological studies evaluating protective dietary patterns for gastric precancerous lesions and gastrointestinal tumors. Emotional status was evaluated by noting experiences of tension, irritability, or depression. We documented the presence of comorbidities and gastrointestinal symptoms, such as reflux esophagitis, peptic ulcers, hypertension, hyperlipidemia, coronary artery disease, acid reflux, heartburn, nausea, vomiting, belching, loss of appetite, and upper abdominal pain. Lifestyle factors, including smoking status, alcohol consumption, physical activity levels, and sleep disorders, were also recorded. Laboratory assessments were performed by collecting 10 mL of fasting venous blood from each participant. The laboratory indicators measured included pepsinogen I (PG I), pepsinogen II (PG II), pepsinogen I/II ratio (PGR), gastrin-17 (G-17), *H. pylori* strain typing, and gene polymorphisms of *PSCA rs2976392*, *PLCE1 rs2274223*, *PRKAA1 rs59133000*, and *MUC1 rs4072037* were analyzed. Additionally, gastroscopic pathological diagnoses were obtained for all patients. This study was reviewed and approved by the Medical Ethics Committee of Tianjin Medical University General Hospital (approval number IRB2019-KY-181), and all participants provided written informed consent.

## 3. Methods

### 3.1. Serological testing

Once the participants completed the questionnaire on risk factors for PLGC, 5 mL of fasting venous blood was collected from those considered eligible for simultaneous testing of PG and G-17 as well as *H. pylori* typing. Serological *H. pylori* typing was performed using a commercial immunoblot assay kit detecting antibodies against urease, cytotoxin-associated gene A (CagA), and vacuolating cytotoxin A (VacA). Type I *H. pylori* infection was defined as positivity for urease antibody together with CagA and/or VacA antibodies, whereas Type II infection was defined as positivity for urease antibody but negativity for both CagA and VacA antibodies.

The reference ranges were as follows: PG: PG I > 70 μg/L and PGR > 3; G-17: 1 to 7 pmol/L; Type I strain: Urease antibody (+), CagA antibody (+), and/or VacA antibody (+); and Type II strain: Urease antibody (+), CagA antibody (−), and VacA antibody (−).

### 3.2. Gene polymorphism detection

We detected the gene polymorphisms of 4 gastric cancer susceptibility loci, namely *PSCA rs2976392*, *PLCE1 rs2274223*, *PRKAA1 rs59133000*, and *MUC1 rs4072037*, by using the TaqMan probe method. DNA was extracted from 5 mL of whole blood stored at −80°C. The process involved primer and TaqMan probe design and synthesis, quantitative PCR amplification, and sequencing.

### 3.3. Statistical methods

Data from the collected questionnaires were entered into Microsoft Excel, and statistical analyses were conducted using SPSS version 26.0 (Chicago, IL) and R software. Continuous variables are expressed as the mean ± standard deviation. For normally distributed data, the *t* test was used for the analysis. For non-normally distributed data, the Mann–Whitney *U* test (rank-sum test) was applied. Categorical variables are presented as counts and percentages, and comparisons between the groups were made using the chi-square test or Fisher exact test. A *P* value <.05 was considered statistically significant. Variables that showed statistical significance in the univariate analysis were subjected to a multivariate logistic regression analysis for identifying independent predictors of PLGC. These predictors were then used to establish the clinical prediction model. The Hosmer–Lemeshow goodness-of-fit test was performed to assess the model’s calibration; a *P* value >.05 indicated a good fit between the predicted and observed values. The model’s clinical predictive value was evaluated by constructing receiver operating characteristic curves, and internal validation was performed using the Bootstrap resampling method.

## 4. Results

### 4.1. Comparison of general and serological indicators

Univariate analysis was conducted to compare general characteristics and serological indicators between the case and control groups. The groups exhibited significant differences in terms of variables such as sex, age, education level, presence of reflux esophagitis, frequency of coarse cereal consumption, levels of PG Ⅰ, PGR, and G-17, *H. pylori* strain type, and hypertension incidence (all *P* < .05). Differences in symptoms including acid reflux, heartburn, and irritability also tended to be significant. However, no statistically significant differences were found between the 2 groups regarding family history of tumors; other dietary habits such as preference for hot, fried, spicy, or high-salt foods; irregular eating patterns; consumption of vegetables, fruits, onions, ginger, garlic, pickles, and dairy products; emotional states such as tension and depression; comorbid conditions and symptoms such as peptic ulcers, incidence of coronary artery disease, hyperlipidemia, nausea, vomiting, belching, loss of appetite, and upper abdominal pain; lifestyle factors including smoking, alcohol consumption, physical activity, and sleep disorders; and laboratory indices such as PG Ⅱ levels (Tables 1–4).

**Table 1:** Comparison of sex, age, education level, and family history between the case and control groups.

General information	Case group	Control group	*P* value
Sex (m/f)	15/23	14/31	<.001
Age (yr, $\$\bar{X}\pm S\$$)	54.71 ± 10.35	41.38 ± 31.95	<.001
Bachelor’s degree and above [example (%)]	10 (26.31)	27 (61.36)	.001
Family history of gastric cancer [cases (%)]	3 (7.89)	2 (4.54)	.866
Family history of other tumors of the GI tract [cases (%)]	11 (28.94)	7 (15.90)	.155

**Table 2:** Comparison of comorbidities and symptoms between the case and control groups.

Comorbid Diseases and symptoms	Number of examples	Case group	Control group	*P* value
Reflux esophagitis	14	12 (31.58)	2 (4.54)	.003
Peptic ulcer	9	5 (13.16)	4 (9.09)	.816
Coronary heart disease	7	4 (10.53)	3 (6.81)	.839
Hypertension	19	13 (34.21)	6 (13.63)	.028
Hyperlipidemia	12	8 (21.05)	4 (9.09)	.224
Acid reflux, heartburn	49	27 (71.05)	22 (50.00)	.053
Nausea, vomiting, belching	23	13 (34.21)	10 (22.73)	.248
Loss of appetite	10	7 (18.42)	3 (6.81)	.109
Upper abdominal pain	14	8 (21.05)	6 (13.63)	.373

**Table 3:** Comparison of dietary habits, living habits, and emotional status between the case and control groups.

Considerations	Number of examples	Case group (n = 38)	Control group (n = 44)	*P* value
Dietary habits				
Hot	25	12 (31.58)	13 (29.55)	.842
Fried	27	10 (26.31)	17 (38.64)	.236
Spicy	14	8 (21.05)	6 (13.63)	.373
High salt	23	12 (31.58)	11 (25.00)	.508
Irregular diet	23	8 (21.05)	15 (34.09)	.190
Whole grain	32	7 (18.42)	25 (56.82)	<.001
Vegetables	62	31 (81.58)	31 (70.45)	.242
Fruits	55	25 (65.79)	30 (68.18)	.818
Onion, ginger, and garlic	62	27 (71.05)	26 (59.09)	.259
Pickles	13	5 (13.15)	8 (18.18)	.353
Living habits				
Smoking	19	10 (26.31)	9 (20.45)	.549
Alcohol consumption	11	6 (15.79)	5 (11.35)	.558
Physical exercise	44	21 (55.26)	23 (52.27)	.787
Sleep disorder	31	17 (44.74)	14 (31.81)	.229
Emotional status				
Tension	42	12 (31.58)	16 (36.36)	.125
Irritability	48	18 (47.37)	30 (68.18)	.056
Depression	32	12 (31.58)	20 (45.45)	.199

**Table 4:** Comparison of serological indexes between the case and control groups.

Serological indexes	Case group	Control group	*P* value
PG Ⅰ (µg/L)	115.47 ± 33.05	161.29 ± 39.02	<.001
PG II (µg/L)	15.56 ± 9.57	17.41 ± 12.63	.546
PGR	9.20 ± 4.34	11.23 ± 4.36	<.001
G-17 (pmol/L)	18.88 ± 11.53	9.25 ± 8.67	.001
*H. pylori* typing (Ⅰ/Ⅱ)	27/11	20/24	.019

G-17 = gastrin-17, *H. pylori* = *Helicobacter pylori*, PG I = pepsinogen I, PG II = pepsinogen Ⅱ, PGR = pepsinogenⅠ/Ⅱ ratio.

### 4.2. Comparison of gene polymorphisms

We analyzed the genotype and allele frequencies of 4 gastric cancer susceptibility loci, namely *PSCA rs2976392*, *PLCE1 rs2274223*, *MUC1 rs4072037*, and *PRKAA1 rs59133000*, in both the case group (patients with PLGC) and the control group (patients without precancerous lesions). The genotype distributions at all 4 loci conformed to the Hardy–Weinberg equilibrium in both groups (Table 5), indicating that the population samples were genetically stable and suitable for association analysis. Although the genotype distributions showed no statistically significant differences between the case and control groups for any of the loci studied (*P* > .05), the analysis of allele frequencies revealed a key finding – the frequency of the A allele at the locus *PSCA rs2976392* was significantly higher in the case group (43.42%) than in the control group (28.41%; *P* < .05). This finding suggests that the A allele of PSCA rs2976392 may be associated with an increased risk of PLGC. By contrast, no significant differences were observed in the allele frequencies at *PLCE1 rs2274223*, *MUC1 rs4072037*, and *PRKAA1 rs59133000* between the 2 groups (*P* > .05 for all; Table 6).

**Table 5:** Hardy–Weinberg equilibrium test.

	*rs2274223*		*rs2976392*		*rs4072037*		*rs59133000*	
Groups	1	2	1	2	1	2	1	2
χ^2^	1.053	0.607	1.469	1.153	0.016	1.550	0.001	0.44
*P* value	.305	.436	.226	.283	.898	.213	.969	.507

Note: Groups: 1 is the case group; 2 is the control group.

**Table 6:** Comparison of genotypes and allele frequencies between the case and control groups.

	Case group (n = 38)	Control group (n = 44)	*P* value
rs2274223			
AA	24 (63.16)	27 (61.36)	.976
AG	11 (28.95)	16 (36.36)	.476
GG	3 (7.89)	1 (2.27)	.504
A	59 (77.63)	70 (79.55)	.765
G	17 (22.37)	18 (20.55)	
rs2976392			
AA	9 (23.68)	5 (11.36)	.139
AG	15 (39.47)	15 (34.09)	.614
GG	14 (36.84)	24 (54.54)	.109
A	33 (43.42)	25 (28.41)	.045
G	43 (56.58)	63 (71.59)	
rs4072037			
AA	26 (68.42)	33 (75.00)	.802
AG	11 (28.95)	9 (20.45)	.372
GG	1 (2.63)	2 (4.55)	1.000
A	63 (82.89)	75 (85.23)	.683
G	13 (17.11)	13 (14.87)	
rs59133000			
CC	11 (28.95)	12 (27.27)	.866
CT	19 (50.00)	24 (54.55)	.681
TT	8 (21.05)	8 (18.18)	.744
C	41 (53.95)	48 (54.55)	.939
T	35 (46.05)	40 (45.45)	

### 4.3. Multifactor logistic regression analysis

The significant variables identified through univariate analysis were included in a binary logistic regression model. The analysis revealed age, PGR, PG Ⅰ, *H. pylori* type (Type I), and whole grain consumption as independent predictors for the risk of PLGC (Table 7).

**Table 7:** Multifactor logistic regression analysis.

Considerations	Regression coefficient	Wald	*P*	OR	95% CI
Age	0.200	7.936	.005	1.221	1.063–1.403
PGR	−0.330	4.224	.040	0.719	0.525–0.985
PG Ⅰ	−0.039	4.841	.028	0.961	0.928–0.996
*H. pylori* typing	2.592	5.230	.022	13.362	1.449–123.252
Whole grains	−2.667	5.176	.023	0.069	0.007–0.691
Constant	−3.854	0.874	.350	0.021	

CI = confidence interval, *H. pylori* = *Helicobacter pylori*, OR = odds ratio, PG I = pepsinogen I, PGR = pepsinogenⅠ/Ⅱ ratio.

### 4.4. Model and evaluation

**Figure 1.**
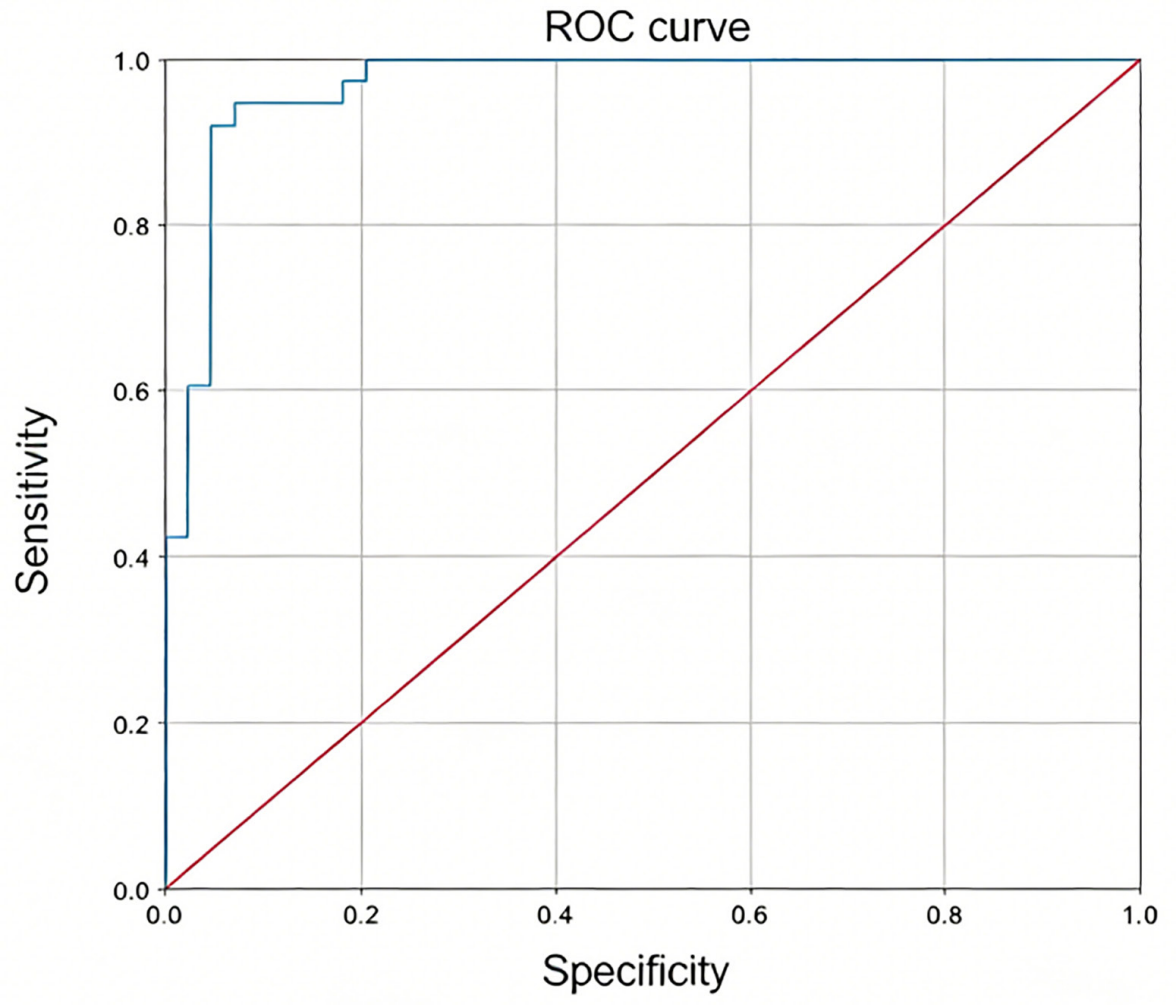
ROC curve for the risk prediction model. ROC = receiver operating characteristic.

**Figure 2.**
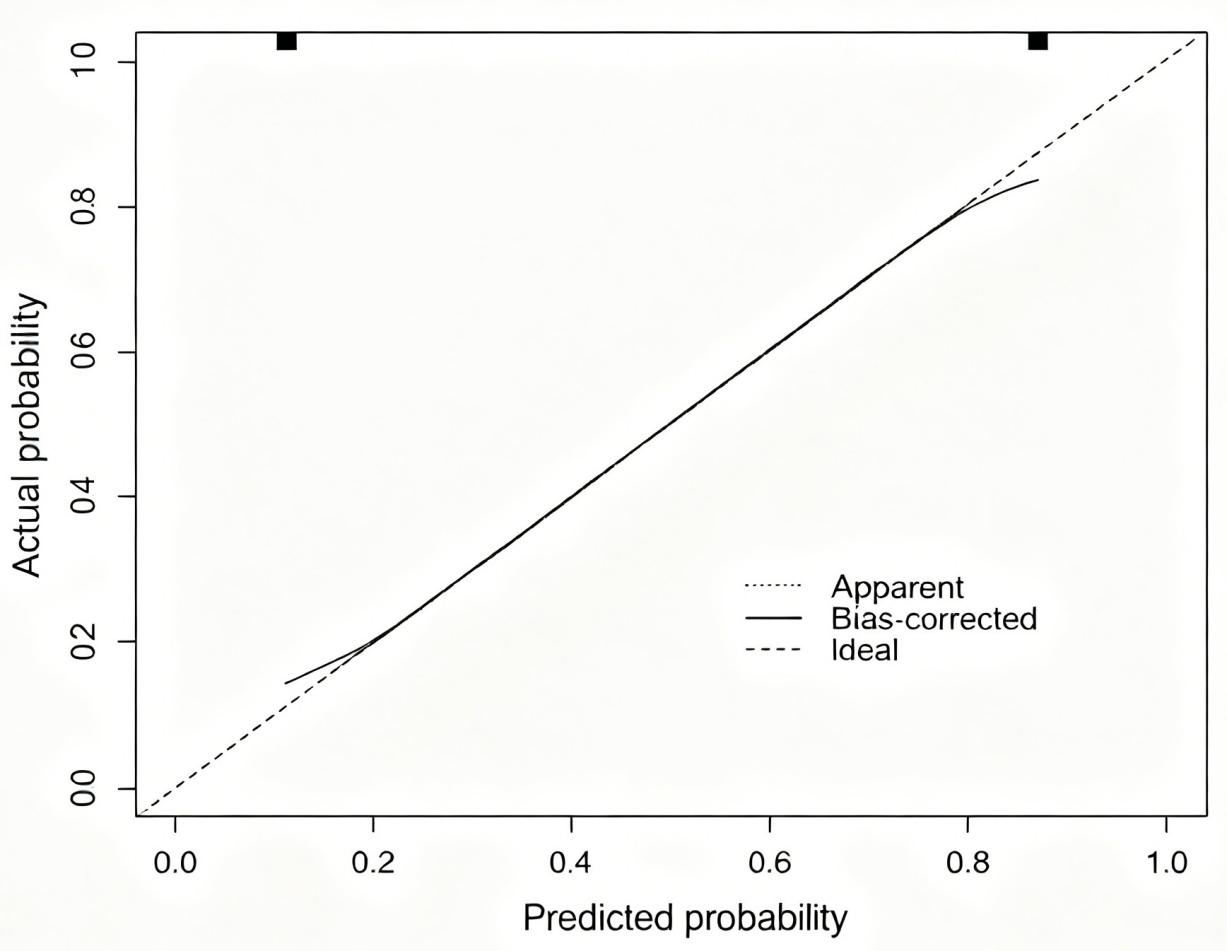
Calibration curve for the risk prediction model.

Based on the results of logistic regression analysis, we developed a risk prediction model for PLGC. The model is formulated as follows: Logit(P) = −3.854 + 0.200 × Age − 0.330 × PGR − 0.039 × PG I + 2.592 × *H. pylori* typing − 2.667 × whole grain, where the unit of age is years, the unit of PG Ⅰ is ng/mL. *H. pylori* type is coded as 1 or 0, with 1 signifying *H. pylori* Type I and 0 signifying Type II. Finally, whole grain consumption is coded as 1 or 0, with 1 indicating frequent consumption (more than twice per week) and 0 denoting infrequent consumption. The Hosmer–Lemeshow goodness-of-fit test yielded a chi-square value of 8.074 (*P* = .426), indicating a good fit between the model and the observed data. To evaluate the predictive performance of the model, we constructed an receiver operating characteristic curve (Fig. 1). The area under the curve was 0.969, and the 95% confidence interval (CI) was in the range 0.935 to 1.000. The optimal cutoff value determined using Youden index was 0.403, corresponding to a sensitivity of 94.7% and a specificity of 93.2%. Internal validation was performed using the Bootstrap method, and the area under the curve was 0.969, which indicated that the results were stable. After model correction (Fig. 2), the observed values closely matched the actual values, which underscored the model’s reliability for predicting PLGC risk in *H. pylori*-positive patients.

## 5. Discussion

Risk prediction models are effective tools for screening high-risk groups in the early disease stage. They help clarify the roles of various risk factors in disease development and prognosis, enabling a focused approach to prevention and the formulation of customized health interventions. However, effective risk monitoring tools for PLGC are currently lacking. In this study, we summarize the relevant factors and characteristics of PLGC. By integrating *H. pylori* typing and the analysis of gastric cancer susceptibility genes, we identified risk factors and constructed a risk prediction model. The factors included in the final model were age, *H. pylori* typing, PG I, PGR, and frequency of whole grain consumption.

Age is an unavoidable risk factor for PLGC, with lesion severity gradually increasing as people age. Studies have found that the incidence of new cases of gastric cancer and its precancerous lesions is highest among individuals aged 50 to 70 years.^[6]^ Liu et al^[7]^ confirmed a positive correlation between age and the incidence of chronic atrophic gastritis, noting that its occurrence increases with advancing age. Similarly, Zhuang et al^[8]^ analyzed gastroscopic and pathological findings from 60,386 patients and discovered a significant age-related increase in the incidence of intestinal metaplasia. European epidemiological studies have also demonstrated a positive correlation between the prevalence of chronic atrophic gastritis and age in both male and female patients, with prevalence rates increasing significantly as age advances.^[9]^ In our study, the mean age of the case group was 54.71 ± 10.35 years, whereas the control group had a mean age of 41.38 ± 31.95 years, with the difference being statistically significant (*P* < .05). This implies that patients with PLGC were significantly older than those without the condition. Multifactorial logistic regression analysis identified age as an independent risk factor for PLGC. As patients age, cellular senescence and decreased self-repairing abilities make it difficult for damaged cells to return to normal after exposure to carcinogens. Additionally, older age is associated with increased cumulative exposure to various risk factors.^[10]^ Thus, age was included as a variable in our risk prediction model.

The World Health Organization classifies *H. pylori* as a Group I carcinogen, and eradication of *H. pylori* reduces the risk of PLGC and gastric cancer itself. Approximately 20% of *H. pylori*-infected patients develop PLGC, but only 2% progress to gastric cancer, a process closely related to host genetics, environmental factors, and the virulence of *H. pylori* strains.^[11]^ The virulence factors cytotoxin-associated gene A (CagA) and vacuolating cytotoxin A (VacA) are crucial for *H. pylori* pathogenicity and act as high-risk factors for the progression of PLGC. Cellular autophagy stabilizes the intracellular environment against *H. pylori* infections, prevents the accumulation of damaged DNA, and inhibits the proliferation of dysplastic gastric cells. However, CagA and VacA can inhibit the activation of upstream signals of autophagy and the maturation of autophagolysosomes in various ways, leading to impaired autophagy in gastric mucosal cells.^[12]^ This impairment hinders the efficient elimination of *H. pylori* through autophagy. Thus, CagA and VacA persist and promote a series of malignant biological processes, such as inflammation, oxidative stress, apoptosis, increased glycolytic activity, and proliferation of dysplastic cells in the gastric mucosa. Type I *H. pylori* strains secrete CagA and/or VacA, and patients infected with these strains have a 2- to 4-fold higher risk of gastric cancer than those infected with nontoxigenic strains.^[13]^ Weng et al^[14]^ found that combining *H. pylori* typing with the detection of the tumor markers CA125 and CA724 can significantly improve the prognostic assessment of gastric cancer. Pang^[15]^ analyzed the effects of different *H. pylori* types on atrophic gastritis and found that Type I strains accelerate the development of intestinal metaplasia, which is closely related to the progression of atrophic gastritis. In our study, the infection rate of Type I *H. pylori* strains was higher than that of Type II strains (57.3% vs 42.7%). Moreover, the infection rate of Type I strains in the case group was significantly higher than that in the control group (71.1% vs 45.4%), with the difference being statistically significant. Therefore, *H. pylori* strain typing was included in the PLGC risk prediction model to provide clinicians with a valuable reference for diagnosis and treatment.

The utility of serum PG levels as markers for gastric mucosal secretory function has been extensively studied. Human PG, a protein-digesting enzyme secreted as a zymogen by chief cells, is classified into 2 types: PG I and PG II. Serum PG levels reflect gastric mucosal atrophy and contribute to risk stratification for individuals at a high risk of gastric cancer; specifically, a low PG I/II ratio (PGR) indicates a higher risk.^[16]^ When gastric mucosal atrophy occurs, the number of fundic gland cells significantly decreases, or they are destroyed due to inflammation, leading to reduced secretion of PG and lower serum PG levels. Serum PG is negatively correlated with the degree and progression of gastric mucosal atrophy. Therefore, PG I, PG II, and PGR can serve as auxiliary diagnostic indices for chronic gastritis and gastric cancer, in addition to gastroscopic biopsy, providing a reference for developing clinical diagnosis and treatment plans.^[17]^ Tong et al^[18]^ demonstrated that PG I levels and PGR decrease significantly with an increase in the degree of atrophy, with the PGR being more indicative. They found that the critical values for severe gastric mucosal atrophy differed significantly between *H. pylori*-positive subgroups (PGR ≤ 9.1 and PGR ≤ 4.5). For the diagnostic value of gastric cancer, the critical value was PGR ≤ 4.7, but in *H. pylori*-negativesubgroups, the cutoff value was PGR ≤ 7.1. In our study, the PGR was 9.20 ± 4.34 in the case group and 11.23 ± 4.36 in the control group, with an odds ratio (OR) of 0.719 in the multivariate regression analysis. The PG I level was 115.47 ± 33.05 μg/L in the case group and 161.29 ± 39.02 μg/L in the control group, with an OR of 0.961. These results indicated that the risk of PLGC increases with decreases in the PGR and PG I levels. Therefore, we included PGR and PG I in our risk prediction model.

Dietary habits are closely linked to the development of PLGC and gastric cancer. Whole grains contain various bioactive components, such as β-glucans, lignans, antioxidants, and phytosterols, which are effective in cancer prevention. Specifically, these components promote apoptosis of cancer cells by increasing the activity or expression of caspase-3 and caspase-9, poly polymerase, apoptotic protease-activating factor-1, cytochrome c, BH3-interacting domain death agonist, Bcl-2-associated X protein, superoxide dismutase, and glutathione. They also induce DNA damage in cancer cells. Additionally, whole grains can inhibit the initiation and metastasis of cancer by affecting the cell cycle and suppress cancer cell proliferation by inhibiting phosphatidylinositol 3-kinase and protein kinase B (Akt) expressions.^[19]^ Xiong et al^[20]^ identified diets devoid of whole grains as a risk factor for PLGC among residents of Nanchang. In our study, multifactorial regression analysis showed that consumption of whole grains was a protective factor against PLGC (OR = 0.069; 95% CI: 0.007–0.69), confirming that regular intake of whole grains can reduce the risk of PLGC. Although whole grains can reduce the risk of PLGC, healthy individuals should pay attention to the frequency and amount of whole grain consumption.

GWASs have identified several loci closely associated with gastric cancer, including *PSCA rs2976392*, *PLCE1 rs2274223*, *MUC1 rs4072037*, and *PRKAA1 rs59133000*. In our study, while the genotype distributions of these 4 gastric cancer susceptibility loci did not differ significantly between the case and control groups, the univariate analysis revealed a significant difference in the frequency of the A allele at *PSCA rs2976392* (43.42% in cases vs 28.41% in controls). A GWAS conducted in Japan and Korea demonstrated that the *PSCA rs2976392 G>A* polymorphism is associated with an increased risk of diffuse gastric cancer, with the A allele enhancing susceptibility.^[4]^ Similarly, Qiu et al^[21]^ found that in the eastern Chinese population, individuals carrying the A genotype at the locus *PSCA rs2976392* had a higher risk of gastric cancer (AG vs GG: OR = 1.61; 95% CI: 1.35–1.91). Lu et al^[22]^ reported that carriers of the GA/AA genotypes at *rs2976392* had a significantly increased risk of gastric cancer compared with those having the GG genotype (OR = 1.37; 95% CI: 1.15–1.62). Furthermore, Qin et al^[23]^ concluded through meta-analysis that PSCA rs2976392 single nucleotide polymorphism is associated with gastric cancer susceptibility in the Chinese population, among whom the Han population with the AA genotype has the highest risk. However, despite these associations, our multivariate analysis indicated no statistically significant difference in the A allele of *PSCA rs2976392*, and therefore, it was not included in the risk prediction model developed in this study.

Using multifactorial logistic regression analysis based on *H. pylori* strain typing, we developed a clinical prediction model for PLGC. Internal validation with the Bootstrap method yielded stable results, enhancing the model’s reliability. Despite these promising findings, our study has several limitations. First, the data were collected from a single center, which may limit the generalizability of our findings to other institutions or populations. Second, the sample size was relatively small compared with those of other risk prediction studies, potentially affecting the statistical power and robustness of our analysis. Third, although Bootstrap internal validation demonstrated stable model performance, external validation was not performed in the present study. Future studies should include temporal validation and geographic validation using independent cohorts from different medical centers and regions. Prospective multicenter validation will be essential to confirm the model’s predictive accuracy and clinical utility across broader populations.

## Author contributions

**Conceptualization:** Huiling Yu, Ping Li, Shujun Gong, Jingwen Zhao, Kui Jiang.

**Data curation:** Huiling Yu, Ping Li, Shujun Gong, Jingwen Zhao, Kui Jiang.

**Formal analysis:** Huiling Yu, Ping Li, Shujun Gong, Jingwen Zhao, Kui Jiang.

**Funding acquisition:** Huiling Yu, Ping Li, Shujun Gong, Jingwen Zhao, Kui Jiang.

**Investigation:** Huiling Yu, Jingwen Zhao, Kui Jiang.

**Writing – original draft:** Kui Jiang.

**Writing – review & editing:** Kui Jiang.
